# Channel Modeling of an Optical Wireless Body Sensor Network for Walk Monitoring of Elderly

**DOI:** 10.3390/s21092904

**Published:** 2021-04-21

**Authors:** Alassane Kaba, Stephanie Sahuguede, Anne Julien-Vergonjanne

**Affiliations:** XLIM Laboratory, UMR CNRS 7252, University of Limoges, 87000 Limoges, France; alassane.kaba@unilim.fr (A.K.); stephanie.sahuguede@unilim.fr (S.S.)

**Keywords:** optical wireless communications, channel modeling, wireless body sensor network, elderly monitoring, mobility

## Abstract

The growing aging of the world population is leading to an aggravation of diseases, which affect the autonomy of the elderly. Wireless body sensor networks (WBSN) are part of the solutions studied for several years to monitor and prevent loss of autonomy. The use of optical wireless communications (OWC) is seen as an alternative to radio frequencies, relevant when electromagnetic interference and data security considerations are important. One of the main challenges in this context is optical channel modeling for efficiently designing high-reliability systems. We propose here a suitable optical WBSN channel model for tracking the elderly during a walk. We discuss the specificities related to the model of the body, to movements, and to the walking speed by comparing elderly and young models, taking into account the walk temporal evolution using the sliding windowing technique. We point out that, when considering a young body model, performance is either overestimated or underestimated, depending on which windowing parameter is fixed. It is, therefore, important to consider the body model of the elderly in the design of the system. To illustrate this result, we then evaluate the minimal power according to the maximal bandwidth for a given quality of service.

## 1. Introduction

In view of demographic trends, the proportion of elderly people in our population is still growing. Aging, even “normally”, is characterized by an increase in frailty, including muscle weakness and instability that can affect level of autonomy. For example, falls are the leading global cause of accidental death and disability in older people, as well as the most common cause of injury and hospitalization [1]. To avoid loss of autonomy, a strategy is to encourage and help older people to be and stay active [2,3].

In this context, Internet of things (IoT), allowing developers to connect multiple devices, systems, and technologies, is increasingly used in particular for monitoring health and physical activity of the elderly through a body sensor network (BSN) [4]. In most cases, the sensors are connected via wireless technologies offering freedom of movement to the wearer especially during activity. In such a remote monitoring context, the wireless BSN (WBSN) system has specific requirements such as high reliability, low power consumption, and high data security. In addition, a WBSN can involve off-body, on-body, and in-body sensors; thus, we can differentiate extra-WBSN and intra-WBSN communication [5]. Connectivity solutions mostly use radiofrequency (RF) wireless technologies through different standards such as Bluetooth Low Energy among others [6,7], with air then being the communication medium. In addition, the human body is also used as a communication medium for intra-WBSN [8]. We focus in this study on wearable on-body sensors with extra-WBSN transmissions.

In the case of extra-WBSN, there are still challenges related to RF spectrum congestion, interference, data security, and privacy. For example, the use of RF technologies may be limited due to potential interference with sensitive devices in environments such as hospitals [9] or due to risks associated with human exposure to RF electromagnetic fields [10]. Other technologies to address these limitations such as optical wireless communications (OWC) have been recently investigated for WBSN applications for both extra-WBSN and intra-WBSN [11,12,13,14,15,16,17].

OWC technology has been explored in several optical bands, covering ultraviolet (UV), infrared (IR), and, more recently, the visible, due to light emitting diode (LED) devices used for both illumination and wireless communications [18,19,20,21]. This technology offers many benefits such as unlicensed spectrum, high bandwidth, simple and cheap front-ends, and no electromagnetic interference. In addition, OWC can enhance indoor communication security and confidentiality as optical rays cannot pass through walls or opaque objects.

One main benefit of OWC for WBSN application is the RF interference reduction near a person wearing a communicating sensor. On the other hand, the presence of the body inducing strong attenuation or blockages of the optical beams is also a challenge, especially when the person is in motion. Another advantage of the OWC is the absence of small-scale fading associated with multipath. A comparative study of the specificities of the optical channel compared to the radio channel was reported in [22]. It highlighted high sensitivity to blocking effects due to the body and a slowly varying behavior of the channel with a relatively long coherence time compared to RF links.

Several studies focusing on OWC channel behavior have highlighted the impact of body and/or movements when designing dynamic WBSN systems [22,23,24,25,26,27,28]. For a realistic modeling in the case of a walking exercise, the overall body movements depend on the mobility scenario including the random path, while the movements of the body parts such as the legs and the arms are more related to the body shape. 

Regarding the scenario for the overall mobility, a simple stochastic model commonly used to describe the behavior of one or more mobile nodes in a confined space is random waypoint (RWP) [29,30]. However, RWP presents some drawbacks, in particular the path discontinuities due to sharp rotations. In order to avoid this issue, some studies have proposed modified models of the RWP (e.g., [22]). Another disadvantage is that the RWP model exhibits a nonuniform spatial node distribution with a high density in the center of the simulation area [30,31]. In the case of our study, we only consider a single mobile node which is the OWC transmitter (Tx) worn by an elderly person during a walk inside a room. The OWC receiver (Rx) is included in a lighting panel at the room ceiling center, which is a classical location for illumination purpose. Consequently, using a RWP model can lead to overestimating the system performance. Thus, we propose to use a different approach, i.e., the random walk model (RW), with a rather uniform spatial distribution of nodes [29,31]. This permits evaluating the communication performance more homogeneously over the entire room area. 

In addition to overall mobility, one challenge is related to body part movement modeling which is linked to the body shape type. In [24,25,26], simple surfaces or volumes only considering body position distribution within the environment were first used to represent the body shape of the person. In [27], a model considering the arm presence and wrist rotations was proposed. However, the arm movement linked to the walk was not considered. New more realistic approaches were recently proposed using 3D human shapes [22,32], which represent a young adult. In [22], the normal walking cycle was broken down into sequences obtained from 3D animation software. These sequences were used to model arm and leg movements during walking. These studies have highlighted that mobility and movement change the geometry of the optical links, which can have an impact on performance.

However, gait pattern is influenced by age and health factors, among others [33]. It is recognized that older people take a different gait than younger people, including slower speed and reduced step length such that the movement of the legs is also reduced [33,34]. In addition, there is also a decrease in the amplitude of the arm swing [35]. Therefore, the question is how these characteristics affect communication performance if the system is designed from a young adult model.

In this article, our contribution is to investigate the behavior of an optical WBSN channel taking into account the specificities of the elderly in relation to the body shape, the arm movement, the step length, and the walking speed. To the best of our knowledge, no study on the performance of OWC-based systems has yet been carried out in the context of monitoring the elderly taking into account parameters related to aging. 

Thanks to the motion-capture recordings available in the literature [36], we define a model of elderly people with particular movements of the limbs during walking. The global mobility scenario is based on the RW model. The behavior of the OWC channel is analyzed from Monte Carlo ray tracing simulations (MCRT) using dedicated software called RapSor [37]. We discuss the interest of a specific model for the elderly, by comparing the characteristics of the channel with those obtained with a classic young person model. Optical WBSN performance for walk monitoring is then dynamically evaluated in terms of the outage probability with an approach taking into account the time correlation during the walk.

The rest of the article is organized as follows: the description of the optical WBSN system, as well as two body models for young people and the elderly, is presented in Section 2. We also define the movements during walking linked to the limbs, the walking speed, and the RW trajectory. In Section 3, the channel statistical analysis is performed, showing the channel gain and delay spread distributions for the two models. Section 4 details the analysis of the performance in terms of outage probability, taking into account the correlation due to walking and compare the results for both models before conclusion.

## 2. System Modeling Description

The studied context is the remote monitoring of a walking elderly person, who is equipped, for example, with an accelerometer and a heart rate sensor. An optical transmitter included in a wrist-worn system transmits data from the sensors to a receiving system located on the room ceiling. Visible wavelengths are not used for communication, as the brightness could be unpleasant to the user’s eyes. Below, we consider transmissions in the infrared (IR) range.

### 2.1. Environnement Description 

The environment is a room of dimensions $6.7\times 6.6\times 3\;m^{3}$ corresponding to the length, width, and height, respectively. This environment does not contain any object except the elements of the system (transmitters Tx and receivers Rx) and the body of the person.

**Figure 1:**
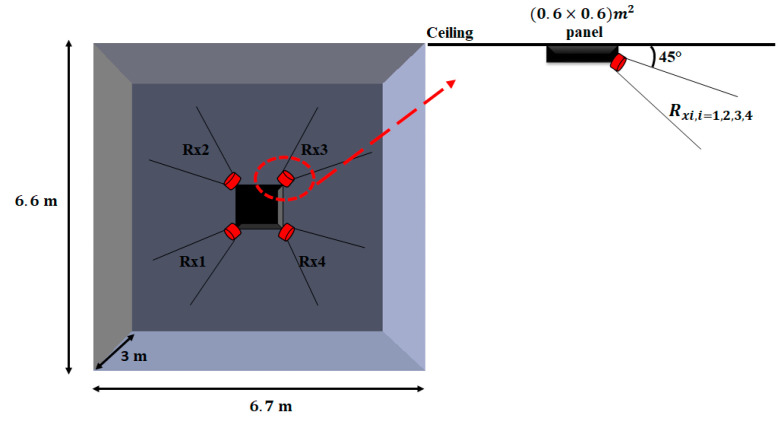
Top view of studied environment and Rx locations.

We assume that a luminaire is located in the center of the room ceiling, designed as a panel of standard dimensions of $0.6\times 0.6\;m^{2}$ and 0.20 m thick, with four identical IR Rxs. According to previously published results [38], all Rx are located at the panel corners oriented at an angle of $45^{{}^{\circ}}$ to the ceiling (see Figure 1). This configuration provides spatial diversity using a switched combining technique that permits optimizing room coverage as the person moves.

The most classical device used as an optical Rx to detect light and convert it into an electric signal is a photodiode. The main properties of photodiodes are their responsivity R (A/W), physical active area $A_{r}\;\left({\mathrm{mm}}^{2}\right)$, and field of view $FOV\;\left({}^{{}^{\circ}}\right)$ [18]. The values of these parameters listed in Table 1 are constant throughout the rest of the paper. 

The Rx orientations represented in Figure 1 are fixed, set to $45^{{}^{\circ}}$, unlike those of the Tx, which vary according to the movements of the person.

We consider that the emitter Tx is an IR light-emitting diode (LED), modeled as a Lambertian source with an order m, linked to the half-power angle $\left(\varphi_{1/2}\right),$ as described in [18].

(1) $$m=\frac{-ln\left(2\right)}{ln\left(cos\left(\varphi_{1/2}\right)\right)}$$

The emitter Tx is even more directional when m is high, i.e., when the half-power angle $\varphi_{1/2}$ is small and vice versa [18]. In addition to the half-power angle, the Tx is characterized by its random location and orientation in the room linked to the random trajectory of the person wearing the sensor as further described. Consequently, the optical line-of-sight (LOS) link condition cannot always be fulfilled. Therefore, the uplink received optical power is due to the power from both LOS and non-LOS paths, i.e., reflected paths over the room surfaces. Due to the roughness of typical indoor environment surfaces that are characterized by a reflectivity parameter ρ, we consider perfect diffuse reflection when the IR beam hits the room surfaces. This latter is consequently modeled using a Lambertian bidirectional reflectance distribution function (BRDF) [37]. In this work, the reflectivity parameter ρ is set to 0.8 for all room surfaces.

### 2.2. Body and Mobility Models 

#### 2.2.1. Body Shape Model 

**Figure 2:**
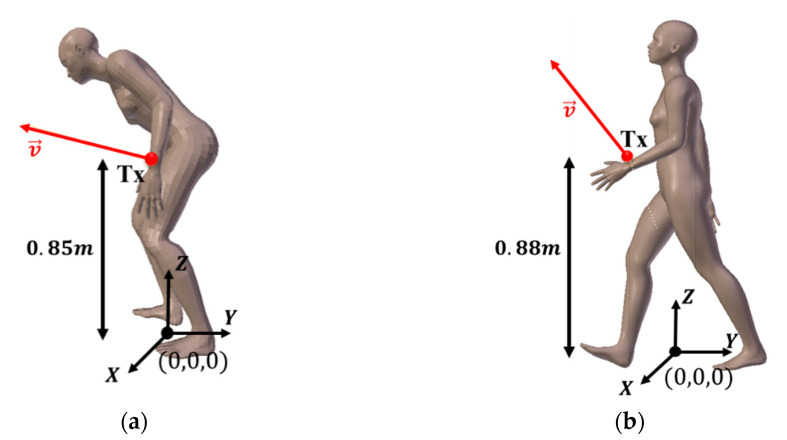
Illustration of 3D human body shape model: (**a**) elderly body shape model; (**b**) young body shape model.

To study the impact of specificities related to age, we consider two types of 3D human body shape model representing a young person with a classical posture (see Figure 2b) and another much older person with the torso bent forward, as illustrated in Figure 2a. Indeed, spinal curvature is a common consequence of aging affecting the quality of walking [39]. 

We can see in Figure 2 that the transmitters located at the wrist are almost at the same height from the floor. The coordinates of the position and orientation vector $\vec{v}$ of Tx from the origin of the coordinate system (0, 0, 0) located between the legs (see Figure 2) are shown in Table 2 for both body models.

These body representations correspond to the starting images of a walking cycle, which can be then modeled using 3D animation software.

#### 2.2.2. Mobility Model Description

In order to consider the mobility, we define two types of movements: the movement of body parts and that of the whole body in the room. Below, we denote local motion for the movement of body parts and global motion for the movement of the whole body.

1. Local Motion

**Figure 3:**
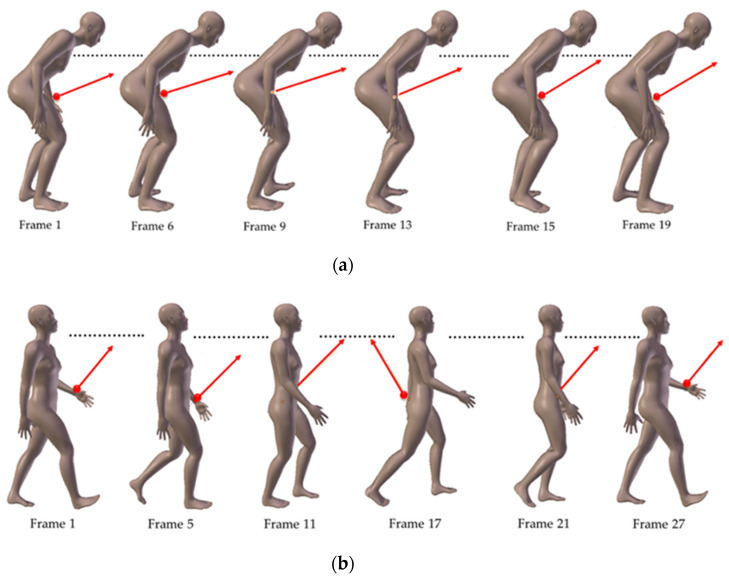
Illustration of walking cycle: (**a**) elderly model; (**b**) young model.

In order to consider movements of arms and legs, we use the 3D modeling software Blender [40] that provides animated bodies of young and old people. These animations are from a database containing real motion captures of a person [36]. They are recorded on different frames constituting a walking cycle, as partially illustrated in Figure 3a for the elderly and in Figure 3b for young people. 

There are differences between both movement patterns. The first concerns the orientation changes of the transmitter at the wrist during the gait cycle. Indeed, we can see in Figure 3a that the orientation vector $\vec{v}$ in the case of the elderly varies but is always in the same direction. On the contrary, for the young model in Figure 3b, the vector may vary in the opposite direction depending on the swing of the arms, for example, between frame 1 and frame 17. This difference is representative of an age-related specificity, which is the decrease in amplitude of the arm swing with aging [35].

The second difference concerns the length of steps, which depends on a person’s height and walking speed, as well as on their age [34,41]. Considering that the young body model is 1.70 m tall and the person walks at a speed of 2 m/s, we assume a step length value of 64 cm [41]. The elderly person walks much slower at a speed of 0.5 m/s. As it is curved, the body height of the elderly person is 1.32 m and we consider that the length of the steps is 20 cm.

2. Global Motion

In addition to local movements, we consider body movements in the room in terms of trajectory. Currently, several mobility models can be used to describe the behavior of one or more mobile nodes in a particular area, the most classic one being RWP [29,30,31]. In the RWP model after a given pause time, a mobile node chooses a destination defined by a random position in the simulation area. The node then travels toward the destination at a speed randomly and uniformly taken in a given range. Once arrived, after a new pause, the process starts again. One characteristic of RWP is that spatial node distribution exhibits a nonuniform behavior mainly concentrated in the center of the area [22], which is a disadvantage in our context. Indeed, since the Rxs are in the center of the ceiling, such a model leads to a nonhomogeneous distribution of the OWC uplinks in the environment, affecting the statistical performance analysis.

**Figure 4:**
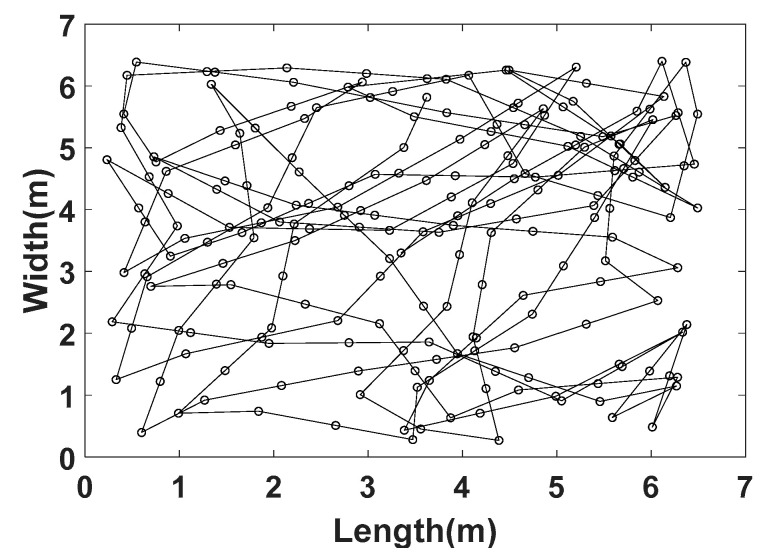
Illustration of a node traveling pattern using RW mobility model.

Therefore, as described in Section 1, we focus in this study on the RW mobility model [29]. With a RW model, each node chooses speed and direction instead of destination, uniformly from preset ranges, and then moves during a constant time interval or over a constant distance. At the arrival location, it remains stable for a certain time and then starts moving again following the same rule. In this work, we consider that the pause time is null. In addition, we use the RW model with a constant travel distance. This distance, called $d_{\mathrm{step}}$, is constant throughout the trajectory, as illustrated in Figure 4, and corresponds here to the average step length, i.e., $d_{\mathrm{step}}=64\;\mathrm{cm}$ for the young person and $d_{\mathrm{step}}=20\;\mathrm{cm}$ for the elderly. 

**Figure 5:**
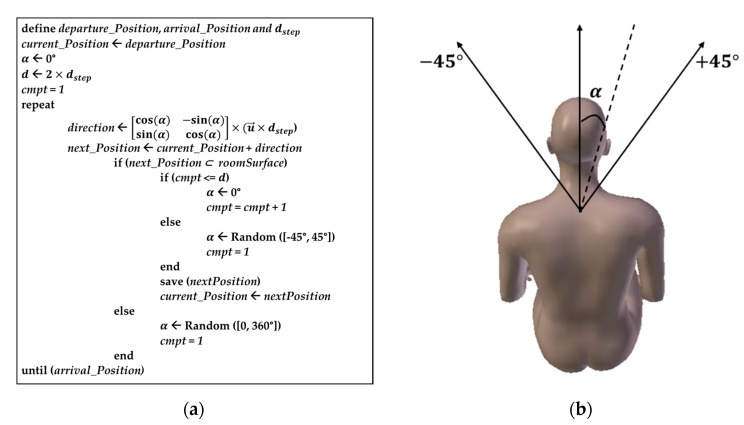
RW trajectory: (**a**) RW trajectory generation algorithm; (**b**) rotation angle for direction change.

**Figure 6:**
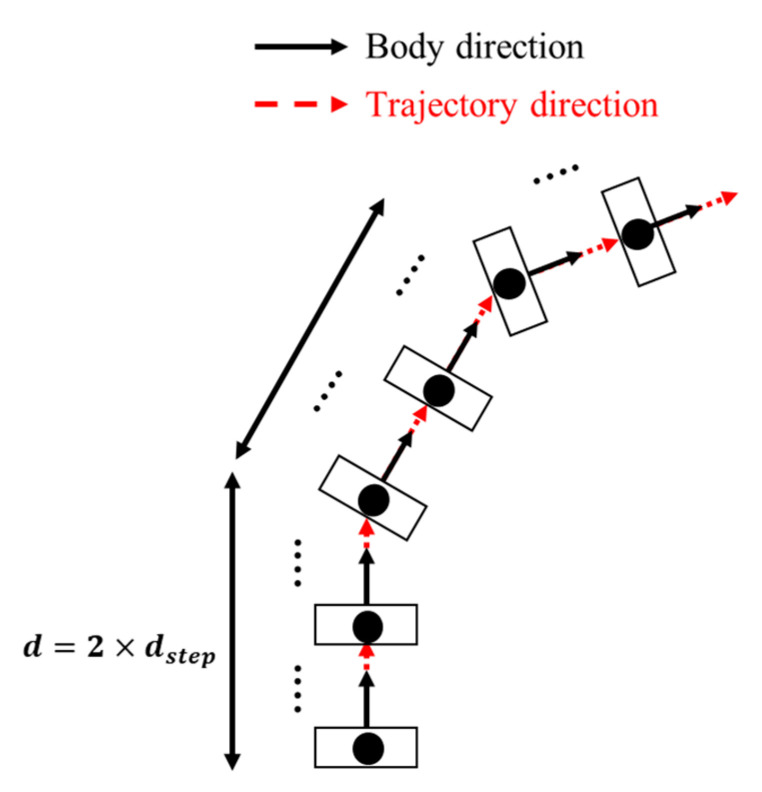
Illustration of direction changes.

We propose in Figure 5a the algorithm for generating the RW trajectory used in this work. In Figure 5a, $\vec{u}$ is a unit vector on the two dimensions (x and y) and α is the rotation angle for direction change, randomly and uniformly chosen between $-45^{{}^{\circ}}$ and $45^{{}^{\circ}}$ as shown in Figure 5b. In cases where the node goes outside the area before the change in direction occurs, we force it to return by choosing a new direction between $0^{{}^{\circ}}$ and $360^{{}^{\circ}}$. Moreover, to synchronize local motions to the global ones, a change in direction is made only after having traveled a certain distance $d=2\times d_{\mathrm{step}}$ corresponding to a walking cycle, as illustrated in Figure 6.

**Figure 7:**
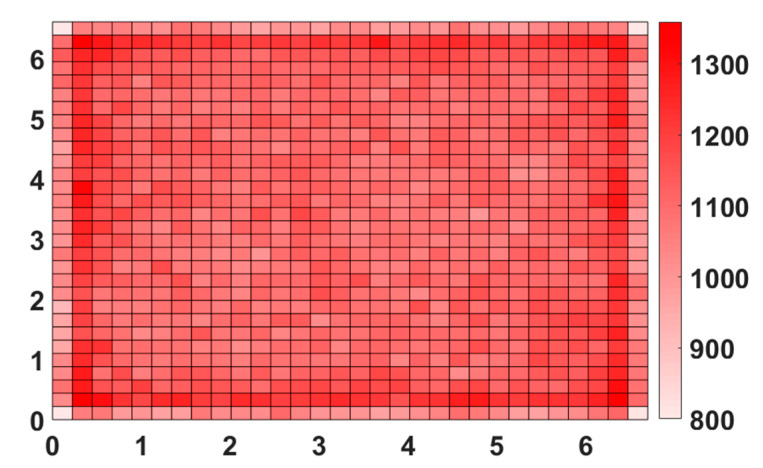
Node distribution for RW mobility.

We represent in Figure 7 the spatial distribution of one million node positions in the studied area for the RW algorithm described above. The node density is represented by the color level. We can verify in Figure 7 that positions of the node are quite homogenously distributed in the room.

## 3. Channel Characterization 

To analyze the optical WBSN channel behavior, we used MCRT-based simulations of channel impulse response (CIR) coupled with the body and mobility modeling that we previously defined.

We used the RaPSor simulation software (Ray Propagation Simulator) developed in our laboratory. It is an extensible tool based on the Netbeans platform and coded in Java, for modeling wave propagation in realistic environments in different frequency domains, from the radio range to the optical one. This simulation software has already been validated for the propagation of IR waves in confined environments and the WBSN context [37,42]. It is based on a stochastic Monte Carlo method, associated with a ray-tracing algorithm, numerically determining both LOS and non-LOS paths contributions (time of arrival and attenuation) from analytical models of reflection and propagation and for one defined link configuration. For all our simulations, in order to manage tradeoffs between the computation time and precision, we consider three reflections per optical beam, which is a classic approach for nondirected transmissions [43]. From this set of optical path contributions, the CIR h(t) is then numerically determined.

Several metrics can be used to characterize the channel, including the DC gain $H_{0}$ and the temporal dispersion of h(t). The DC gain is one of the most important features representing the ratio between the optical received power $P_{r}$ and the emitted one $P_{t}$. It is defined by the Fourier transform of impulse response taken in f=0, and it is linked to h(t) by the following expression [18]:

(2) $$H\left(0\right)=\int_{-\infty }^{+\infty }h\left(t\right)dt=H_{0}$$

Since h(t) is a discrete signal, numerically determined from the ray-tracing technique, $H_{0}$ is obtained in this case via a discrete summation.

The temporal dispersion of h(t) allows assessing the impact of inter-symbol interference (ISI). Thus, another main channel parameter is the root-mean-square (RMS) delay spread $\tau_{RMS}$ defined as follows [18]:

(3) $$\tau_{RMS}=\sqrt{\frac{\int_{0}^{+\infty }{\left(t-\tau_{0}\right)}^{2}{\left|h\left(t\right)\right|}^{2}dt}{\int_{0}^{+\infty }{\left|h\left(t\right)\right|}^{2}dt}}$$

The mean excess delay $\tau_{0}$ is expressed by

(4) $$\tau_{0}=\frac{\int_{0}^{+\infty }t{\left|h\left(t\right)\right|}^{2}dt}{\int_{0}^{+\infty }{\left|h\left(t\right)\right|}^{2}dt}$$

When taking into account the mobility of the person in the room, we must consider the link established, which is a set of impulse responses h(t) resulting from local and global movements, as defined in Section 2. Therefore, the channel metrics such as DC gain and RMS delay have to be statistically analyzed, and the channel behavior is characterized using the statistical distribution.

We first obtained from MCRT simulation results the sets of $H_{0}$ and $\tau_{RMS}$ values for both young and elderly body shape along the generated trajectories with the movements associated with age. In this work, we assume that the two bodies are characterized by a reflectivity value ρ equal to 0.1 that corresponds to a very absorbent material. In addition, we consider for the channel analysis 10,000 CIR values corresponding to different Tx positions and orientations when the person moves inside the room. 

**Figure 8:**
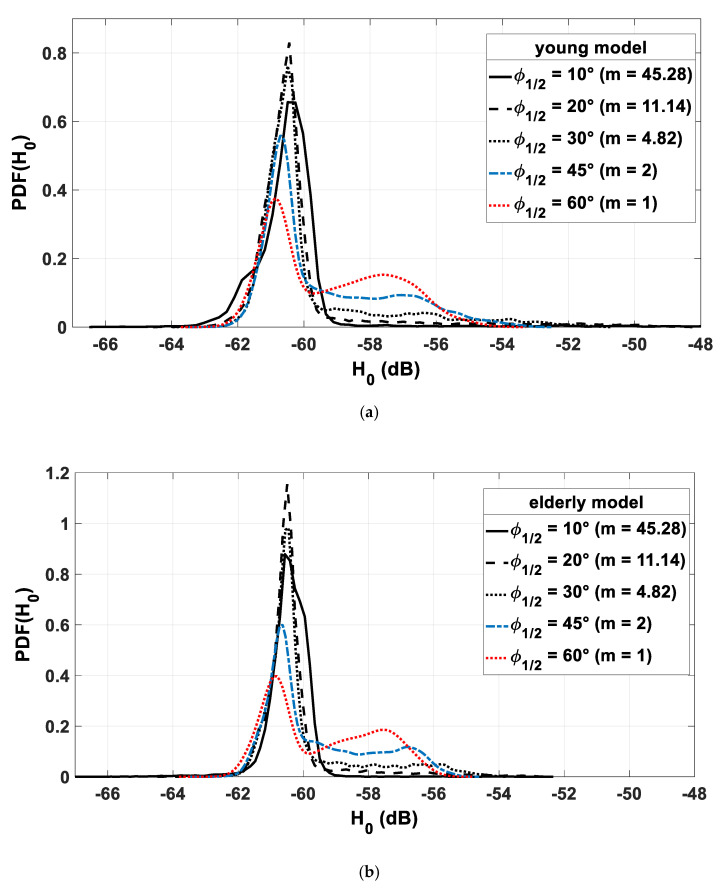
PDFs of $H_{0}$ for different values of $\varphi_{1/2}$ with young model (**a**) and with elderly model (**b**).

We analyze channel behavior on the basis of the optical gain probability density function $pdf\left(H_{0}\right)$. In Figure 8, the PDFs for the two body models of young (Figure 8a) and elderly (Figure 8b) people are plotted for different Tx directivity characterized by half-power angle values $\varphi_{1/2}$ (corresponding to certain m values).

First, we observe that the distribution of the gain values for the two models follows the same behavior depending on the directivity of the transmitter Tx.

Indeed, it can be noted that, for both models, low $\varphi_{1/2}$ values lead to a narrow PDF, traducing a most probable value around −60.4 dB. More precisely, it is for $\varphi_{1/2}=20^{{}^{\circ}}$ that the PDF is the narrowest for both models, and this is even more the case for the elderly one. For higher $\varphi_{1/2}$ values, it can be observed that the spreading range increases and differs between the two models. Actually, gain values are up to −50 dB for the young model and −54 dB for the elderly one, with $\varphi_{1/2}=45^{{}^{\circ}},\;60^{{}^{\circ}}$ being the angles corresponding to highest probability in this range. If we focus on the other part of the curves, i.e., the lowest gain values, we can observe that gain values are more dependent on the $\varphi_{1/2}$ value for the young model than for the elderly one. This is linked to the arm movements, which are more important in the case of the young model, introducing more cases where the optical link is weakened. On the other hand, for both models, the Tx angle value $\varphi_{1/2}=45^{{}^{\circ}}$ is the optimal one, leading to the same highest minimal gain value (around −61 dB).

Therefore, as a conclusion, $\varphi_{1/2}=45^{{}^{\circ}}$ is a good tradeoff to optimize optical gain values for both models; thus, we consider this optimal angle below.

Lastly, it can be noted that, for this angle, the maximum channel gain value for the young body is −53 dB, while, for the elderly, it is 2 dB lower, around −55 dB. Thus, using a young body model, the channel behavior could be overestimated when designing the WBSN system for monitoring the elderly.

**Figure 9:**
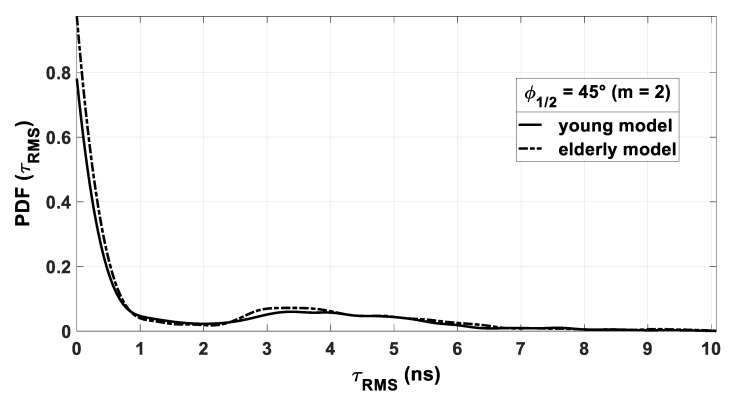
PDF of $\tau_{\mathrm{RMS}}$ with young and elderly models.

Moreover, to study the impact of the model on the channel behavior in terms of delay, in Figure 9, the PDF of $\tau_{RMS}$ is plotted for the optimal Tx angle of $45^{{}^{\circ}}$. We see that, in this case, the choice of a model has very little impact as the two curves are quite identical.

We report in Table 3 the maximum delay spread $\tau_{\mathrm{RMS}\_\mathrm{MAX}}$ and the corresponding maximal bandwidth $B_{\mathrm{MAX}}$ for avoiding ISI considering both models. Indeed, when the delay spread is significantly shorter than the symbol period $T_{S}$, the ISI effect can be neglected. Thus, the maximal bandwidth $B_{\mathrm{MAX}}=\frac{1}{T_{S}}$ estimated from $\tau_{\mathrm{RMS}\_\mathrm{MAX}}$ is such that

(5) $$B_{\mathrm{MAX}}\leq \frac{1}{10\tau_{{\mathrm{RMS}}_{\mathrm{MAX}}}}$$

From the results in Table 3, we assume that channel delay dispersion and, hence, ISI effect are roughly negligible for a bandwidth up to approximately 10 MHz, which is compatible with the rates of most sensors dedicated to health or activity monitoring, generally lower than 1 Mbps [12]. 

Since the proposed optical WBSN system concerns walk monitoring, it must be able to transmit data with the greatest reliability regularly during exercise. For this aim, we develop in the next section an approach taking into account channel behavior evolution along the trajectory to assess the performance and compare the impact of both channel models for young and elderly persons. 

## 4. Performance Evaluation

### 4.1. Metric Definitions

Linked to the optical channel gain obtained in the previous section and depending on the modulation, the signal-to-noise ratio (SNR) is a key metric used to assess performance, taking into account the power of the transmitter and the noise contribution. The SNR γ can be defined in a general manner as follows [18]:

(6) $$\gamma =\frac{P_{t}{}^{2}H_{0}{}^{2}R^{2}}{\sigma^{2}}$$

where $\sigma^{2}$ represents the total variance of the noise assuming additive white Gaussian noise (AWGN), $P_{t}$ is the average emitted optical power, and R is the responsivity of the photodiode.

For indoor environments, induced background shot noise and receiver thermal noise are generally the dominant noise sources [44]. However, the most limiting factor is shot noise related to the induced ambient current $I_{B},$ as described in [18].

(7) $$\sigma^{2}=2qI_{B}B$$

where q is the electron quantum charge, and B is the bandwidth of the modulated signal. In our context, we use a value of 200 μA for $I_{B}$ as classically reported for indoor environments [32,45].

The channel gain is a random variable as seen in Section 3; thus, so is the SNR. 

The chosen performance metric in this analysis is the outage probability $P_{out}$, classically defined as the probability that γ becomes lower than a given performance corresponding to a γ limit value called $\gamma_{0}.$

(8) $$P_{out}=p\left(\gamma <\gamma_{0}\right)=\int_{-\infty }^{\gamma_{0}}pdf\left(\gamma \right)d\gamma$$

For a given value of $\gamma_{0}$, $P_{out}$ can be, thus, obtained using the channel gain distribution linked to the person positions in the room.

**Figure 10:**
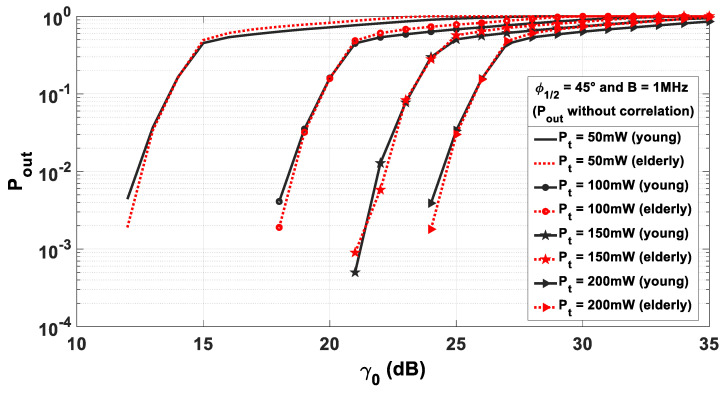
Uncorrelated outage probability $P_{out}$ as a function of $\gamma_{0}$ for different emitted power.

We report in Figure 10 the evolution of $P_{out}$ as a function of $\gamma_{0}$ for the models of young and old people considering various average emitted power $P_{t}$ and a bandwidth B of 1 MHz. We consider $P_{t}$ values between 50 mW and 200 mW taking into account the power constraint due to IR eye-safety considerations [46]. To construct an accurate analysis, we considered 10,000 values of γ issued from the walking scenario described in Section 2.

We observe that the $P_{out}$ curves are similar for both models whatever the emitter power. This is in accordance with the results shown in Section 3 regarding channel gain PDF.

We could therefore conclude that the specificities linked to age have no impact. However, this statistical approach considers all the positions without taking into account the followed trajectory. In order to determine the performance of the transmission continuously during the walk, the correlation between successive positions must be taken into account. Indeed, as the person walks, the optical gain and, thus, the SNR γ and the performance vary. By considering the walking speed, we can study the evolution of the performance either as a function of time or as a function of the traveled distance.

**Figure 11:**
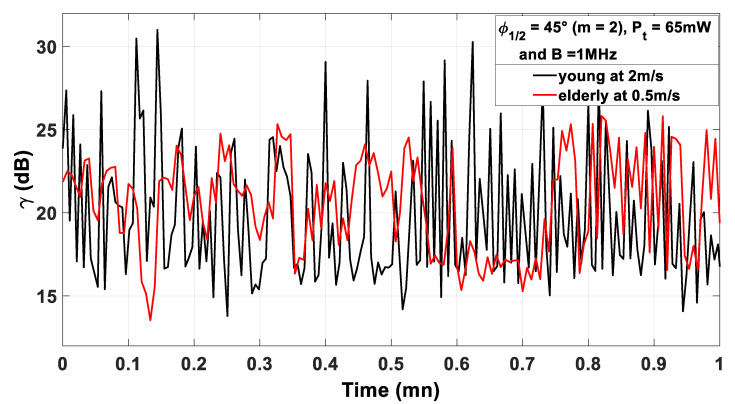
γ as a function of time for an example of RW trajectory during 1 min.

As an example, we report in Figure 11 the evolution of γ over 1 min considering both young and elderly models, as defined in Section 2, with $P_{t}=65\;\mathrm{mW}$ and B=1 MHz. In addition, as described in Section 2, the models of the young and elderly include the walking speed, which was set to 2 m/s and 0.5 m/s, respectively.

This example shows that performance varies over time for the two models. Actually, we observe that γ undergoes strong variations over time whatever the body model. However, these variations are more important for the young person than for the elderly. This is linked to the differences between the two models in terms of velocity and local movements. 

To account for correlation between the successive values of γ corresponding to consecutive positions along the trajectory, we developed an analysis based on the sliding window technique. The principle is to evaluate the performance taking into account its variation over time by using two parameters: the size of the observation window and the value of the sliding step, related to the overlap between each window.

In the analysis below, the window overlap for the sliding process is chosen equal to the person step length $d_{step}$.

To evaluate $P_{out}$ considering a sliding window, we only focus on windows where γ is initially greater than $\gamma_{0}$. If γ becomes lower than $\gamma_{0}$ at least once over a selected window, we consider that as a case of outage. The outage probability is then obtained by dividing the number of cases of outage by the total number of observation windows over the entire trajectory.

The observation window size is an important parameter for the analysis. Indeed, this represents the interval during which the system reliability must be ensured with a given outage probability criteria. We denote *T* as the time duration of the window, whereas D is the corresponding distance, related to the walking speed $S_{W},$ varying with age.

(9) $$D=S_{w}\times T$$

Below, we compare performance in terms of $P_{out}$ as a function of *T* and *D* for both models.

### 4.2. Outage Performance with Sliding Windowing

The outage probability $P_{out}$ taking into account correlation was evaluated as a function of $\gamma_{0}$ for the models of young and old people considering an average emitted power $P_{t}$ of 65 mW and a bandwidth B of 1 MHz. 

**Figure 12:**
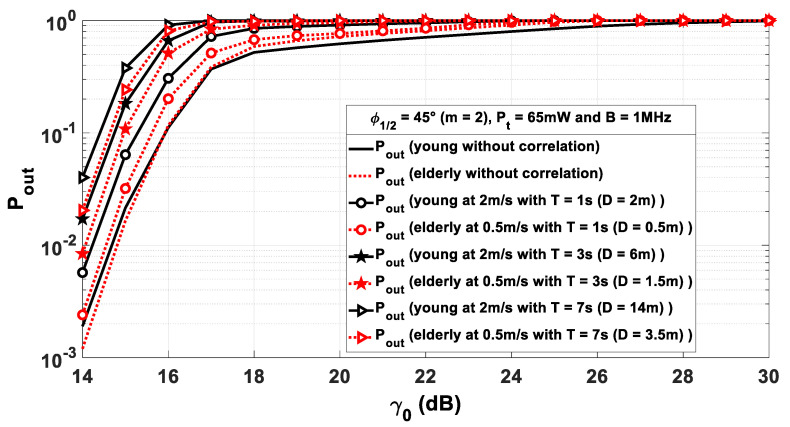
Outage probability $P_{out}$ as a function of $\gamma_{0}$ for different window sizes in terms of time duration T.

For both models, results are plotted in Figure 12 considering given window sizes T equal to 1 s, 3 s, and 7 s.

In addition, we report the uncorrelated outage probability curves for the same power for comparison.

The sliding step corresponds to a length $d_{\mathrm{step}}=0.64\;m$ for the young person and $d_{\mathrm{step}}=0.2\;m$ for the elderly. Considering the used walking speeds for young and elderly people, this corresponds to a step duration of 0.32 s and 0.4 s, respectively.

First, it is noted that, considering the evolution along the trajectory, the performances are degraded in comparison to the uncorrelated case. This is all the more significant with the size of the sliding window. Thus, it is important to take into account correlation for performance evaluation.

In addition, we see that performance between the two models diverges. It is always better with elderly model. Thus, including specificities of age with the elderly model allows not underestimating the WBSN performance.

**Figure 13:**
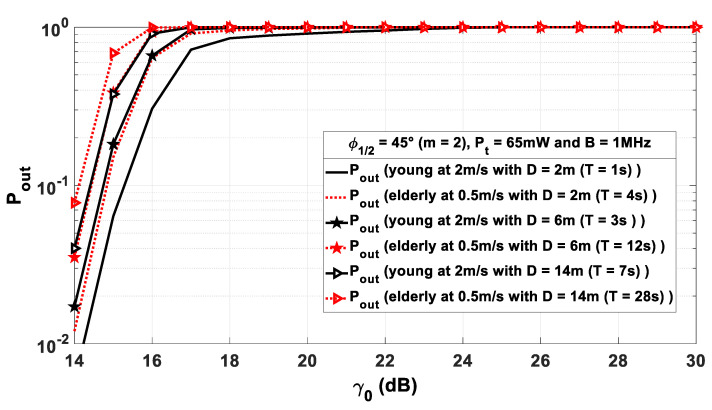
Outage probability $P_{out}$ as a function of $\gamma_{0}$ for different window sizes in terms of distance duration *D*.

To complete the analysis, Figure 13 shows the performance by fixing the size of the observation window in terms of distance *D.* Here, we observe that, as *D* increases, the performance degrades. In this case, whatever *D* value, and contrary to the previous analysis, performance is overestimated when using the young model instead of the elderly.

In conclusion, when considering a young body model and depending on the chosen windowing parameter (time or distance), the evaluation of correlated performance along the walking path is either overestimated or underestimated. Therefore, it is more appropriate to use a model incorporating the elderly specificities in terms of walking speed and step length in order to properly design the optical WBSN system for transmission during person’s walking. 

**Figure 14:**
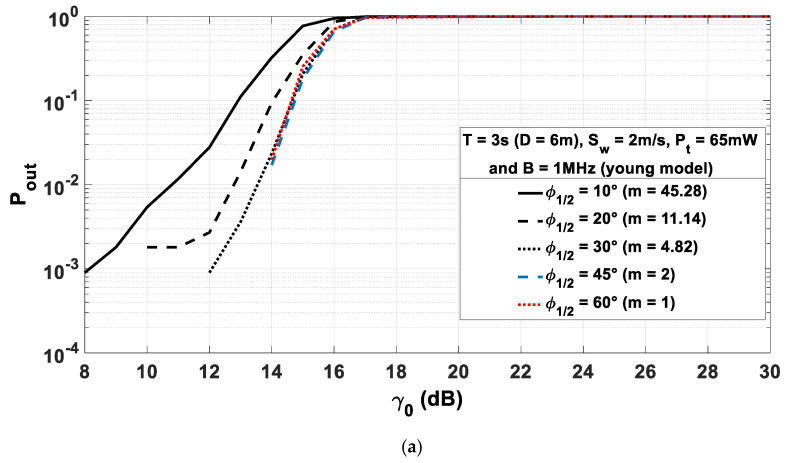

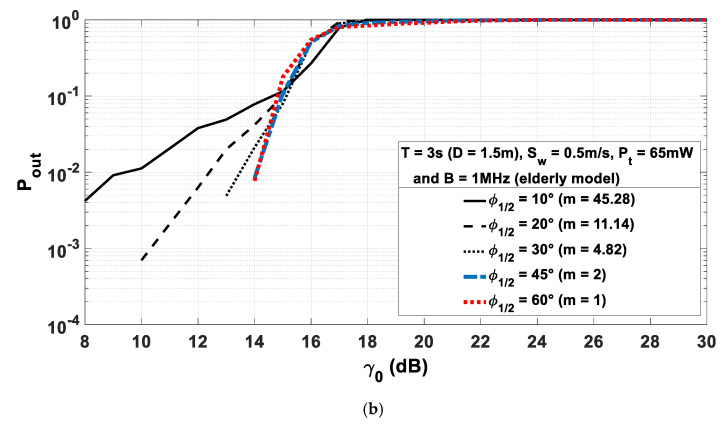
Outage probability $P_{out}$ as a function of $\gamma_{0}$ for different values of $\varphi_{1/2}$: with young model (**a**) and with elderly model (**b**).

The results presented above were obtained with a half-power angle of the transmitter $\varphi_{1/2}=45^{{}^{\circ}}$, which was shown as the optimal angle in Section 3 when considering a statistical analysis of $H_{0}$ without considering the correlation. Thus, in order to study the impact of the correlation approach we used, in Figure 14, the outage probability $P_{out}$ is plotted for the young body model (Figure 14a) and the elderly one (Figure 14b) for different values of $\varphi_{1/2}$ and a window size T=3 s.

First, we observe in Figure 14a for the young body model that, regardless of the outage probability, the best performance is obtained for half-power angles greater than $30^{{}^{\circ}}$. On the other hand, for the elderly body model in Figure 14b, when the outage probability is high (greater than $10^{-1}$, i.e., for $\gamma_{0}$ greater than 15 dB), performance is quite insensitive to the value of the directivity of the transmitter. Instead, when the required quality of service increases, imposing outage probabilities lower than $10^{-1}$, we see that the half-power angles corresponding to the best performances are 45° and $60^{{}^{\circ}}$. 

Thus, when the correlation is taken into account, for both models, the optimal $\varphi_{1/2}$ can always remain $45^{{}^{\circ}}$.

In the next subsection, we investigate the performance related to emitted power and bandwidth for both models.

### 4.3. Performance of WBSN Related to Power and Bandwidth

The WBSN system for walk monitoring is based on a sensor worn by a person and communicates via OWC. Thus, the emitted power is a main concern due to the lifetime of the body sensor system, as well as the eye-safety limitation when using IR [46]. In this context, it is, therefore, preferable to minimize the optical emitted power.

Considering a target value of $\gamma_{0}$ and a given value of sliding window parameter (T or D), we investigate the minimal emitted power $P_{tmin}$ required to satisfy the quality of service in terms of outage probability $P_{out}$. This power value depends on the bandwidth B related to the transmission rate.

**Figure 15:**
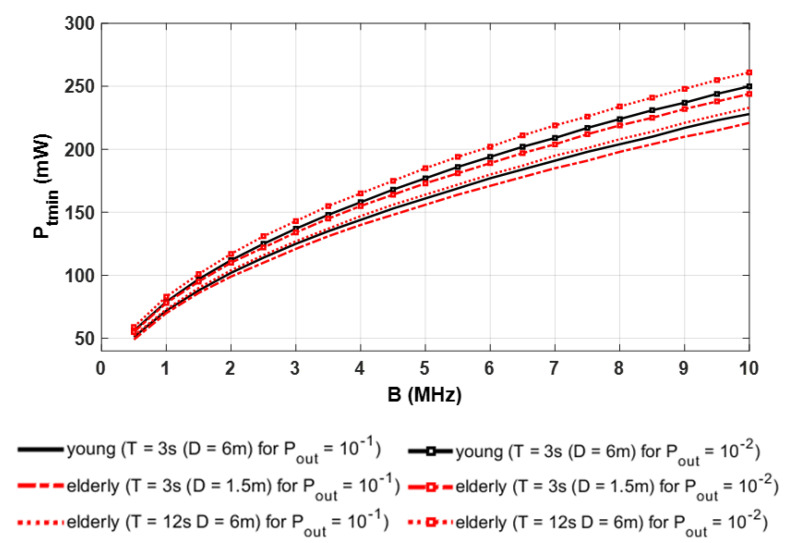
Evolution of emitted power as a function of bandwidth for $\gamma_{0}=15.6\;\mathrm{dB}$ with $\varphi_{1/2}=45^{{}^{\circ}}\;\left(m=2\right)$.

As an example, for both channel models corresponding to young and elderly persons, Figure 15 shows the evolution of $P_{tmin}$ as a function of bandwidth B until 10 MHz for a given $\gamma_{0}$ of 15.6 dB and two targets $P_{out}$ values of $10^{-1}$ and $10^{-2}$. Actually, it has been verified that a packet failure of 10% is a maximum tolerable value [47].

The value $\gamma_{0}=15.6\;\mathrm{dB}$ for the most basic OWC modulation, i.e., on–off keying, corresponds to a bit error rate of $10^{-9}$, which is a classical metric for medical WBSN [10].

Moreover, we can consider results in Figure 15 for different window sizes in time and distance T=3 s and 12 s and D=1.5 m and 6 m. As expected, the required minimal power for a target $P_{out}$ increases with bandwidth regardless of the channel model and parameters of sliding windows. In addition, the required power is obviously higher when the quality of service increases, i.e., $P_{out}$ diminishes. As the minimal optical power is always lower than 250 mW, we can also conclude that eye-safety conditions are respected for IR using sources with low Lambertian order [10].

By comparing the two models, we verify in Figure 15 that the use of a channel model with a young person’s body leads to slightly overestimating or underestimating the power required. Indeed, considering the results for $P_{out}=10^{-1}$ and B=5 MHz as an example, we can see that the minimum power is 156 mW for the elderly body model when T=3 s and 164 mW when D=6 m, whereas it is around 161 mW for the young body model for T=3 s and D=6 m.

On the other hand, we remark that the difference is more significant in terms of the maximal bandwidth to ensure a given $P_{out}$ when the power is fixed. If the emitted power is set to 200 mW, for example, using a young body model leads to a maximal bandwidth of 6.5 MHz for $P_{out}=10^{-2}$, whereas it is either 23% lower (equal to 5 MHz) using the elderly model with D=6 m or 7% higher (equal to 7 MHz) with T=3 s.

## 5. Conclusions

In this article, we studied the behavior of an optical WBSN channel for walk monitoring of elderly, taking into account age-related specificities in relation to body shape, arm swing, step length, and walking speed. From gait recordings for an elderly person, we modeled the movements of the limbs and considered a particular body model, different from that of a young person. In addition, we considered the movement of the whole body in the room following a random trajectory using an RW mobility model.

To study the impact of age-related specificities, the behavior of the optical WBSN channel was analyzed when using the proposed model or that based on a young body model. Simulations based on an MCRT method provided the impulse responses of the optical links between the transmitter worn on the wrist of the elderly or young person and a reception system on the ceiling.

Numerical results on channel gain and delay spread statistics determined from the impulse responses were presented. We first analyzed the results globally and without taking into account the evolution of the person’s walk. In this case, we concluded that, by using a young body model, the gain of the channel could be overestimated when designing the WBSN system for monitoring the elderly. On the other hand, the choice of a young or elderly model had very little impact on the selectivity of the channel.

Then, the performance was evaluated in terms of outage probability by taking into account the channel evolution along the random path using the sliding window technique. Considering an optimal transmitter half-power angle of 45°, we first verified that the performance degrades compared to an analysis without correlation. The correlation effect was studied for different sizes of sliding windows expressed in time or distance. By comparing the outage probability results for both models, we showed that, when considering a young body model, the performance along the walking path is either overestimated or underestimated. This conclusion also applies to determining the minimum transmit power and bandwidth required for a given quality of service.

Consequently, it is suitable to use a model incorporating the elderly specificities for designing the optical WBSN system for regular monitoring during walking.

## Figures and Tables

**Table 1:** List of Rx parameters.

	Definition	Values
	Positions [X Y Z] (m)	$R_{X1}\;:\left[3.0\;3.05\;2.8\right]$ $R_{X2}\;:\left[3.0\;3.65\;2.8\right]$ $R_{X3}\;:\left[3.6\;3.65\;2.8\right]$ $R_{X4}\;:\left[3.6\;3.05\;2.8\right]$
Receivers	Orientation angle (°)	45
	Physical active area A_r_ (mm^2^)	34.5
	Field of view FOV (°)	45
	Responsivity R (A/W)	1

**Table 2:** Tx positions and orientation vector coordinates on a young and elderly body.

	Position [*X Y Z*] (m)	Normalized Orientation $\mathrm{vector}\;\vec{v}$[*x y z*]
Young	[0.22 0.14 0.88]	[0.75−0.38 0.53]
Elderly	[0.26 0.05 0.85]	[0.64−0.74 0.14]

**Table 3:** Maximum delay spread $\tau_{\mathrm{RMS}\_\mathrm{MAX}}$ and maximum data rate.

	$\tau_{RMS\_MAX}\left(ns\right)$	$B_{MAX}\;\left(MHz\right)$
Young	9.90	10.09
Elderly	10.08	9.92

## Data Availability

Not applicable.
